# Maternal mitochondrial function affects paternal mitochondrial inheritance in *Drosophila*

**DOI:** 10.1093/genetics/iyae014

**Published:** 2024-01-30

**Authors:** Jinguo Cao, Yuying Luo, Yonghe Chen, Zhaoqi Wu, Jiting Zhang, Yi Wu, Wen Hu

**Affiliations:** Department of Basic Medicine, Gannan Medical University, Ganzhou 341000, China; Key Laboratory of Mitochondrial Medicine, Gannan Medical University, Ganzhou 341000, China; Department of Basic Medicine, Gannan Medical University, Ganzhou 341000, China; Department of Public Health and Health Management, Gannan Medical University, Ganzhou 341000, China; Department of Basic Medicine, Gannan Medical University, Ganzhou 341000, China; Department of Basic Medicine, Gannan Medical University, Ganzhou 341000, China; Key Laboratory of Prevention and Treatment of Cardiovascular and Cerebrovascular Diseases, Gannan Medical University, Ministry of Education, Ganzhou 341000, China; Key Laboratory of Genetic and Developmental Related Diseases, Gannan Medical University, Ganzhou 341000, China; Key Laboratory of Prevention and Treatment of Cardiovascular and Cerebrovascular Diseases, Gannan Medical University, Ministry of Education, Ganzhou 341000, China

**Keywords:** paternal mitochondria inheritance, mtDNA, *mt:CoⅠ*, *Drosophila*, mitochondria transmission

## Abstract

The maternal inheritance of mitochondria is a widely accepted paradigm, and mechanisms that prevent paternal mitochondria transmission to offspring during spermatogenesis and postfertilization have been described. Although certain species do retain paternal mitochondria, the factors affecting paternal mitochondria inheritance in these cases are unclear. More importantly, the evolutionary benefit of retaining paternal mitochondria and their ultimate fate are unknown. Here we show that transplanted exogenous paternal *D. yakuba* mitochondria can be transmitted to offspring when maternal mitochondria are dysfunctional in *D. melanogaster*. Furthermore, we show that the preserved paternal mitochondria are functional, and can be stably inherited, such that the proportion of paternal mitochondria increases gradually in subsequent generations. Our work has important implications that paternal mitochondria inheritance should not be overlooked as a genetic phenomenon in evolution, especially when paternal mitochondria are of significant differences from the maternal mitochondria or the maternal mitochondria are functionally abnormal. Our results improve the understanding of mitochondrial inheritance and provide a new model system for its study.

## Introduction

It is widely accepted that mitochondria are inherited maternally. Indeed, mechanisms preventing paternal mitochondrial transmission to offspring in spermatogenesis and post-fertilization have been reported (Wallace 2007; Levine and Elazar 2011; Sato and Sato 2017). In *C. elegans*, paternal mitochondria are identified post-fertilization, degenerated in a CPS-6 (endonuclease G)-mediated pathway (Zhou *et al.* 2016), and then encapsulated by autophagosomes and delivered to lysosomes (Al Rawi *et al.* 2011; Sato and Sato 2012). In *D. melanogaster*, most of the paternal mitochondrial DNA (mtDNA) is degraded by the endonuclease G-mediated pathway during spermatogenesis, and the remaining paternal mtDNA is removed via cellular remodeling during spermatid tail formation (DeLuca and O’Farrell 2012). After fertilization, the paternal mitochondria are poly ubiquitinated, surrounded by MVB (multivesicular body)-like vesicles and finally degraded through autophagy (Politi *et al*. 2014).

Compared with *D. melanogaster* and *C. elegans*, the mechanism of paternal mitochondria degradation pathway is more complex in mammals. In mice, paternal mitochondria are poly ubiquitinated during sperm formation before entering the egg (Sutovsky *et al.* 1999). Shortly after fertilization, paternal mitochondria are recognized and surrounded by autophagy-related factors (Al Rawi *et al.* 2011). It remains unclear whether paternal mitochondria are eliminated during embryogenesis. Some studies suggest that paternal mitochondria are not eliminated, but rather unevenly distributed to daughter cells during embryonic mitoses (Luo *et al.* 2013), while others report that paternal mitochondria are cleared by mitophagy and/or the proteasome system (Rojansky *et al.* 2016; Song *et al.* 2016). Although detailed mechanisms remain to be elucidated, it is clear that various pathways work to ensure that maternal inheritance of mitochondria occurs in animals.

Despite the numerous pathways that either eliminate paternal mitochondria or prevent their replication, paternal mitochondrial leakage is still detected in many species such as fish (Peng *et al.* 2018), insects (Kondo *et al.* 1990; Ma *et al.* 2014; Polovina *et al.* 2020), mice (Gyllensten *et al.* 1991; Kidgotko *et al.* 2013), sheep (Zhao *et al.* 2004), and even human beings (Luo *et al.* 2018). One study found that paternal mitochondria in mice are eliminated in intraspecific crosses but not in interspecific crosses (Kaneda *et al.* 1995). Another group hypothesized that the elimination of paternal mitochondria postfertilization is mainly due to the combined effects of a nuclear coding factor that recognizes sperm mitochondria and another factor in eggs (Rokas *et al.* 2003). Given the same genetic background during intraspecific hybridization, the two factors are thought to coordinate to eliminate paternal mitochondria, whereas the genetic background variation during interspecific hybridization weakens the elimination process and leads to paternal mitochondrial inheritance. However, paternal mitochondrial leakage during intraspecies hybridization has been detected in *Drosophila* (Matsuura *et al.* 1991; Nunes *et al.* 2013), which indicates that the genetic background difference cannot entirely explain all cases of paternal mitochondrial leakage.

Paternal mitochondrial inheritance in humans is highly controversial. The first report of paternal mitochondrial leakage in humans was published in 2002 (Schwartz and Vissing 2002). In 2018, a study was published reporting a high degree of mitochondrial heteroplasmic variants in 17 individuals from 3 unrelated families, and the authors proposed that the inheritance of paternal mitochondria is similar to autosomal dominant inheritance (Luo *et al.* 2018). This work created significant controversy and has been intensely debated in the field. Indeed, it has since been argued that there is insufficient evidence to confirm paternal mitochondrial inheritance in humans from that study, mainly because the mitochondrial heteroplasmic types in the offspring could not be established in their father or grandfather (Lutz-Bonengel and Parson 2019). Yet another study sequenced the mitochondrial DNA from 41 patients with mitochondria diseases, as well as their parents, and found no case supporting paternal mitochondrial inheritance (Rius *et al.* 2019). Moreover, the analysis of 33,105 whole genome sequences revealed that the rare inherited nuclear-encoded mitochondrial segments (NUMTs) can create the impression of heteroplasmy, similar to paternal mitochondrial inheritance (Wei *et al.* 2020). Consequently, there remains little supporting evidence for paternal mitochondrial inheritance in humans.

Although paternal mitochondrial inheritance has been reported in several species, the question of whether paternal mitochondria can be systematically inherited has been debated for decades. The controversy remains ongoing due to the lack of effective ways to detect paternal mitochondria and measure the efficiency of paternal mitochondrial inheritance. We thus do not know under which circumstances paternal mitochondria are preserved, or if there is a mechanism regulating paternal mitochondria retention. It is also important to discern whether the preserved paternal mitochondria are functional, as there would be little apparent evolutionary benefit from retaining nonfunctional mitochondria. Previously, there have been a few studies showing paternal mitochondrial leakage in fruit flies and mice (Kidgotko *et al.* 2013; Wolff *et al.* 2013; Polovina *et al.* 2020), but no studies showing the functional state of the retained paternal mitochondria.

The ability to select offspring with paternal mitochondria, track them, and reveal the genetic mechanism of paternal mitochondria transmission is a challenging task. In our study, as a first step toward this goal, we developed a research model to efficiently select offspring with paternal mitochondria. In this model, we utilized two *D. melanogaster* strains. One is a temperature-sensitive (29°C) lethal mtDNA mutant strain, designated *mt: CoⅠ^ts^*, which contains a point mutation in the mitochondrial gene *CoⅠ* (Hill *et al.* 2014). The other strain is a heteroplasmic fly (*Het*) strain with two kinds of mitochondria, the proportionally minor *D. melanogaster* mitochondrion (*mt: CoⅠ^ts^*, *mt: nd2^del1^*) and *D. yakuba* mitochondrion (Lieber *et al.* 2019). We crossed the *mt: CoⅠ^ts^* (female) with *Het* (male) and recovered F1 survivors at restrictive temperature. Subsequent analyses confirmed successful paternal transmission of functional mtDNA to offspring. Interestingly, F1 survivors were similarly recovered from crosses of *mt: CoⅠ^ts^* females with different wild type *melanogaster* strains, although definite evidence for the presence of paternal mtDNA in the offspring remains elusive. Our results nevertheless indicate that paternal mitochondrial inheritance might be a regulated process, and this study established a genetically tractable model for its study.

## Materials and methods

### Fly genetics

The fly strains used in this study are CS, *w^1118^*, *mt: CoⅠ^ts^* (gifted by Dr. Xu Hong), *Het* fly strain (gifted by Dr. Thomas R. Hurd). Flies were raised in an approximate 12 h light (700 lux)/12 h dark cycle, at temperatures of 18°C or 29°C.

### Genetic crosses

**Fig. 1.**
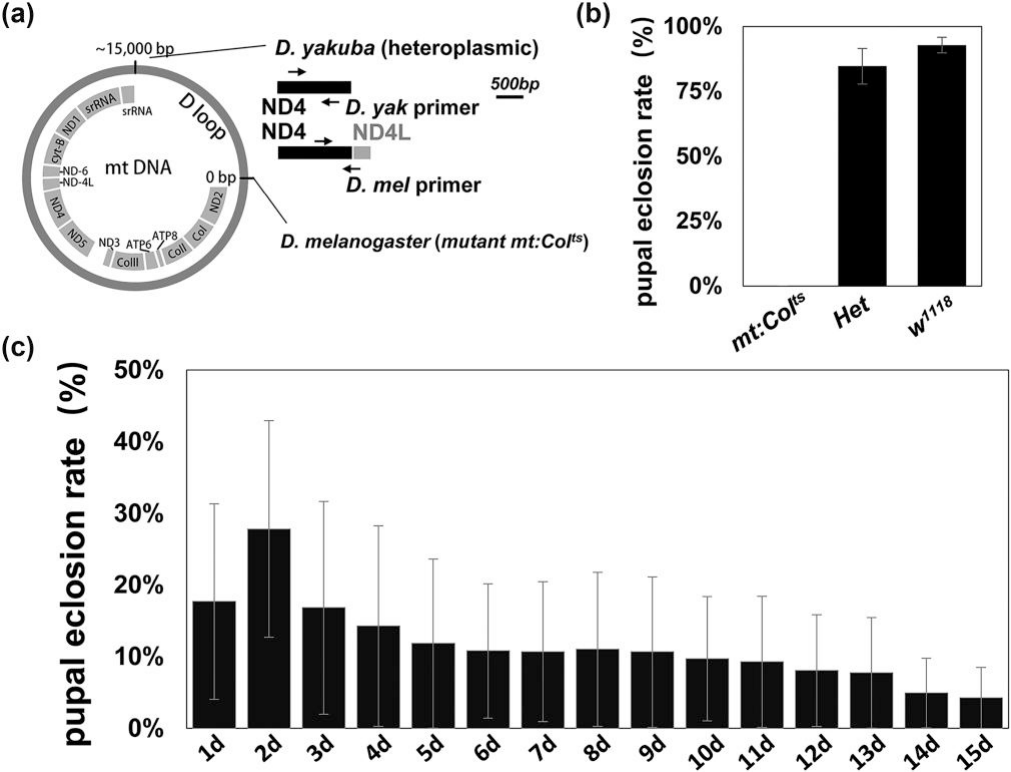
The pupal eclosion rate from the cross *mt: CoⅠ^ts^* females with *Het* males correlates with the age of female flies. a) Schematics of the mitochondrial genome and the D loop regions of *D. yakuba* and *D. melanogaster.* PCR primers specifically targeting the unique regions of *D. yakuba* and *D. melanogaster* mtDNA sequences are shown. b) The pupal eclosion rates of *w^1118^*, *mt: CoⅠ^ts^*, and the *Het* flies. The results shown are mean ± SE. c) The pupal eclosion rate of F1 flies from the cross of *mt: CoⅠ^ts^* females (ages, 1–15 days) with *Het* males. The results shown are mean ± SE.

**Fig. 2.**
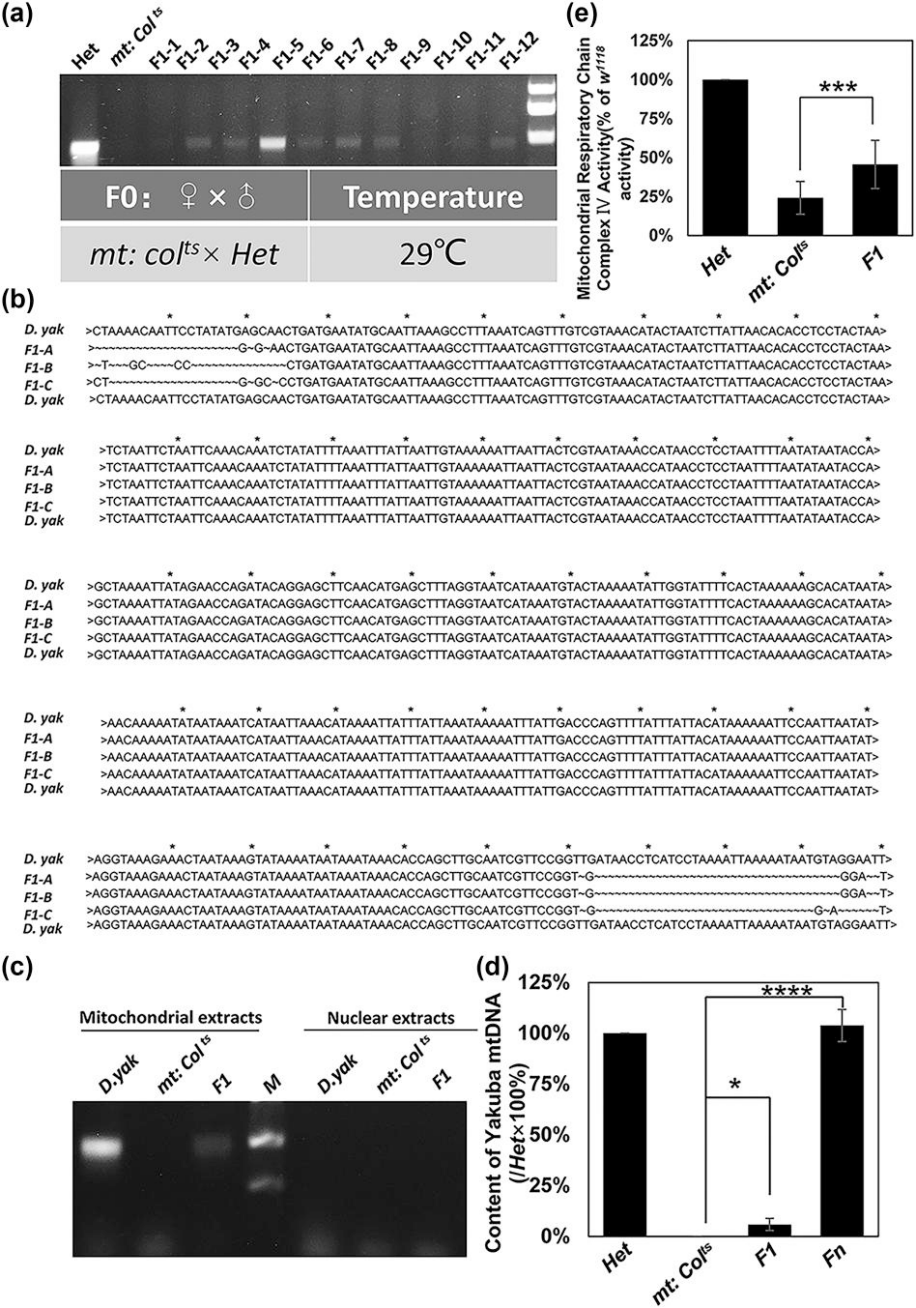
Detection of paternal mtDNA in F1 flies from the cross *mt: CoⅠ^ts^* females with *Het* males. a) Paternal *D. yakuba* mtDNA can be detected in F1 flies from the cross *mt: CoⅠ^ts^* females with *Het* males. Amplified DNA fragments from mtDNA of *mt: CoⅠ^ts^* and *Het* are used as negative and positive controls. b) Amplified DNA fragments of F1 flies from the cross *mt: CoⅠ^ts^* females with *Het* males are shown aligned with *D. yakuba* mtDNA sequence. c) Mitochondrial and nuclear extracts from *mt: CoⅠ^ts^*, *Het*, and F1 flies were separated and amplified with *D. yakuba* specific primers. Amplified fragments can be detected in the mitochondrial extracts from *Het* and F1 flies, but not in the nuclear extracts. d) Real-time RT–PCR analysis of mtDNA content. d*) yakuba* mtDNA in F1 flies is about 5.85% and in Fn (*n* > 30) is about 103.76% of *Het* flies. Four data sets were averaged. The content of *D. yakuba* mtDNA in F1 (*P < 0.05) and Fn (****P < 0.0001) flies is significantly different from that in *mt: CoⅠ^ts^* flies. e) Mitochondrial Respiratory Chain Complex Ⅳ Activity of *Het*, *mt: CoⅠ^ts^* and F1 flies at 29°C. F1 flies acquire about 45.67% complex Ⅳ activity of wild-type flies. The results shown are mean ± SE. Six data sets were averaged. The complex Ⅳ Activity in F1 flies is significantly different from that in *mt: CoⅠ^ts^* flies (***P < 0.001).

**Fig. 3.**
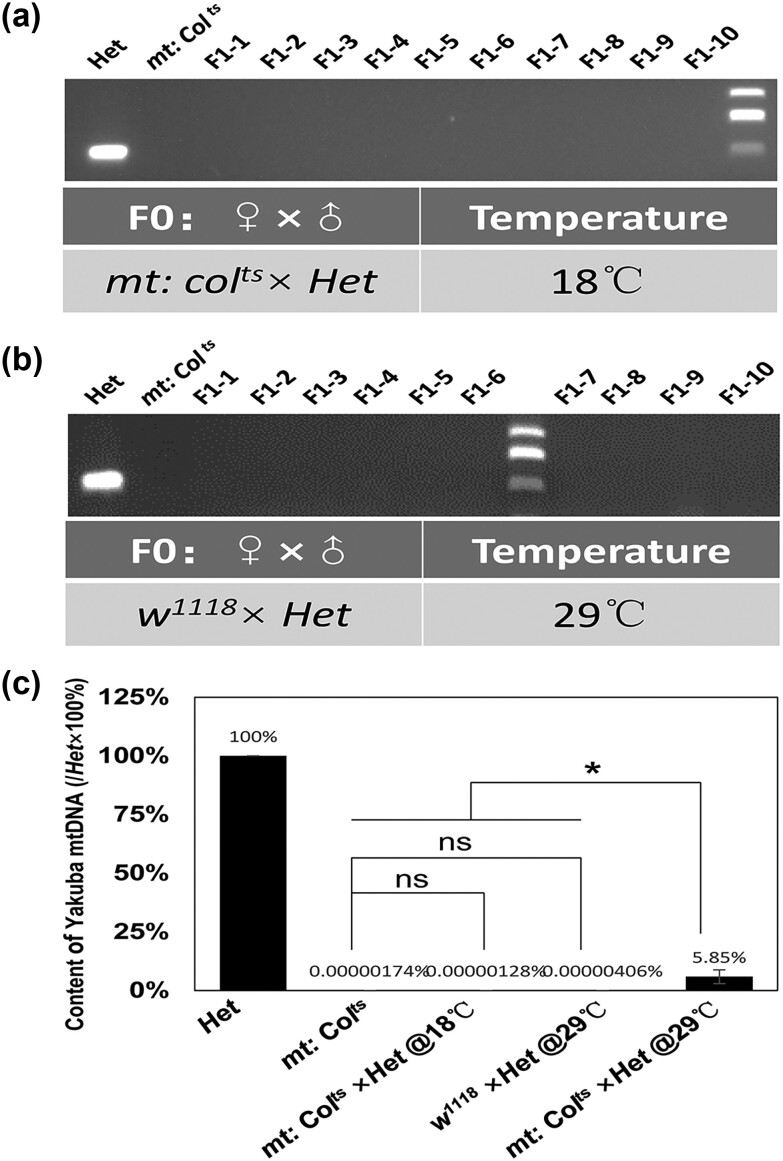
Detection of paternal mtDNA in F1 flies from control crosses. a) Detection of paternal *D. yakuba* mtDNA in F1 flies from the cross *mt: CoⅠ^ts^* females with *Het* males at 18°C. Amplified DNA fragments from mtDNA of *mt: CoⅠ^ts^* and *Het* flies are used as negative and positive controls. No amplified bands could be detected from *mt: CoⅠ*^*ts*^ × *Het* F1 flies cultured at 18°C. b) Detection of paternal *D. yakuba* mtDNA in F1 flies from the cross *w^1118^* females with *Het* males at 29°C. Amplified DNA fragments from mtDNA of *mt: CoⅠ^ts^* and *Het* flies are used as negative and positive controls. c) Real-time RT–PCR analysis of mtDNA content. The content of *D. yakuba* mtDNA in F1 flies from *mt: Col^ts^* females ×*Het* males at 18℃ and *w^1118^* females ×*Het* males at 29℃ has no significant difference to that in *mt: Col^ts^* flies. Three data sets were averaged.

**Fig. 4.**
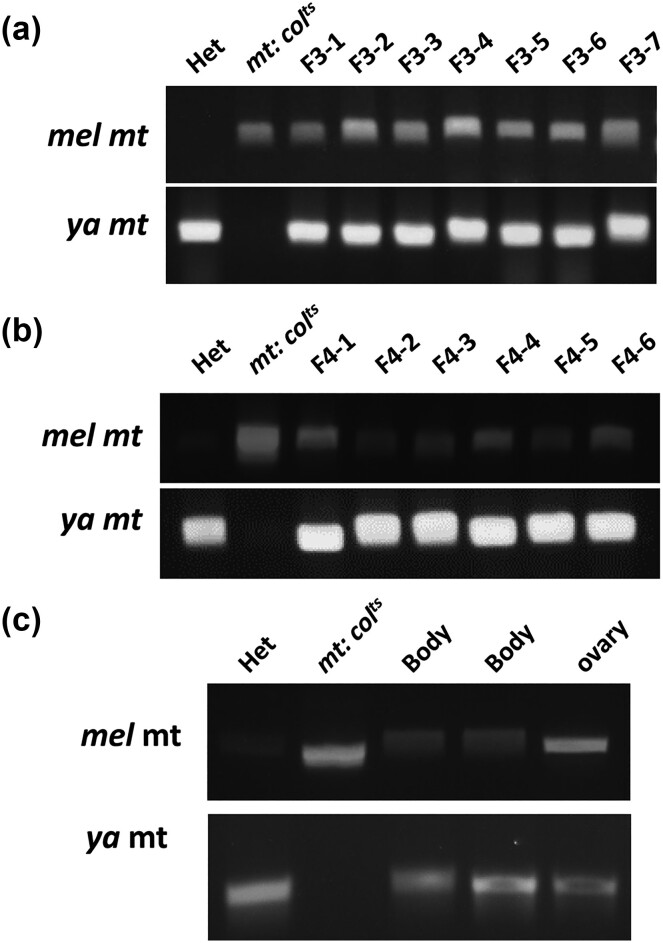
Detection of paternal mtDNA in the F3, F4 generations and the bodies/ovaries of F3 flies. a) Detection of paternal *D. yakuba* mtDNA in F3 flies. Amplified *D. yakuba* DNA fragments from *mt:CoⅠ^ts^* and *Het* flies were used as negative and positive controls. b) Detection of paternal *D. yakuba* mtDNA in F4 flies. Amplified DNA fragments from *mt:CoⅠ^ts^* and *Het* flies were used as negative and positive controls. c) Detection of *D. yakuba* mtDNA in the bodies and ovaries of F3 flies. Dissected ovaries and bodies from F3 flies were separately subjected to DNA extraction. Amplified *D. yakuba* DNA fragments were detected in F3 ovaries and bodies.

The crosses of F0 were conducted at 18℃, then the eggs were collected within 12 hours, and transferred to 18℃ (control group in Fig. 3a) or 29℃ for culturing until pupal eclosion (Fig. 1b and c, Fig. 2, Fig. 3b, Fig. 4, Supplementary Fig. 1). The F1 pupal eclosion rate of each vial was counted and the average rate of each group and condition was calculated. The mean values are presented. The cross of F1 females with *mt: CoⅠ^ts^* males was also conducted at 18℃, then the eggs were collected within 12 hours and were transferred to 29℃ until pupal eclosion. The subsequent generations after F1 were kept at 29℃ for culturing for their life-time.

### Mitochondrial respiratory chain complex Ⅳ activity assay and statistical analysis

For each experiment, 50 4-day-old flies from every group were collected and homogenized in 1 ml mitochondrial extracting buffer and analyzed using the Mitochondrial Respiratory Chain Complex Ⅳ Activity Assay Kit (D799473, Sangon Biotech). The enzyme activity for each group was calculated following the standard formula recommended in the kit. Each data point was the average of at least 3 independent experiments. The mean values and SE of each group are presented. The *P* values were determined with t-tests, as implemented in Prism 8 for Windows 10 GraphPad Software.

### DNA extraction, PCR, and restriction enzyme treatment

Individual flies were homogenized in 100 μL solution A (0.1M Tris, 0.1M EDTA, 0.1%SDS, pH 9.0) and incubated at 70℃ for 25 min for cell lysis and DNA denaturation. 14 μL KAc (8M) was added for renaturation on ice for 30 min. DNA extracts were separated at 4℃ by centrifugation for 15 min at 14,000 rpm. After that, total DNA was precipitated with 60 μL isopropyl alcohol and washed with 70% ethanol, then finally diluted in 10 μl ultra-pure water. 2 μL DNA extract was used for each PCR reaction (*Taq* DNA polymerase, M0273L, NEB).

For enzyme treatment in Supplementary Fig. 1b and c, 25 μL PCR product was purified and treated with XhoI restriction enzyme (XhoI, R0146S, NEB) at 37℃ overnight.

The separation of mitochondria and nucleus in Fig. 2c was conducted following the protocol from the Qproteome Mitochondria Isolation Kit (QIAGEN, 37612). About 30 1-day-old flies were homogenized and subjected to the mitochondria isolation process. The pellets containing mitochondria and nuclei were later subjected to the DNA extraction process. Resultant pellets containing mtDNA or nuclear DNA were suspended in 30 μL water. PCR was carried out using 2 μL mtDNA and nuclear DNA extraction.

### qPCR quantification of mtDNA

One-day-old flies were homogenized and subjected to DNA extraction. The resultant pellet was suspended in water (10 μL ultra-pure water/1 single fly). qPCR was carried out using 1 μL of DNA extraction and 400 nM of each primer pair with a SLAN-96P Real-time PCR machine and GoTaq qPCR Master Mix (2X) (Promega, A6001). The PCR program was: 2 min at 50℃, 5 min at 95 °C, 40 cycles of 95 °C for 30 s and 60 °C for 1 min. Dissociation curves were generated through a thermal denaturation step and used to verify amplification specificity. The data were calculated by the ΔΔCt method and *rp49* was used as the reference gene. The *P* values were determined with t-tests. The data were normalized to wild-type samples and plotted as fold changes.

### PCR primers

The primers binding specifically to mtDNA of either *D. melanogaster* or *D. yakuba* are:

*D. Yakuba*-mito-F1 (5′-GTTAATAAACCAGCTAAAACAATTCCTATATGA-3′),
*D. Yakuba*-mito-R1 (5′-AGGTTATCAACCGGAACGATTG-3′),
*D. Melanogaster*-mito-F (5′- GACCCAGAAACTGGAGCTTC −3′)
*D. Melanogaster*-mito-R (5′- GAGTATGTGAAGGGGCCTTA −3′)rtt

The primers for amplification of mtDNA fragments including the XhoI restriction site in *D. melanogaster* are:

F1 (5′-GAGGAGATCCTATTTTATATCAAC-3′)
R1 (5′-GTGTAAGCATCTGGGTAATC-3′)

The primers used in qPCR are:

*rp49*-F (5′-TCCTACCAGCTTCAAGATGAC-3′)
*rp49*-R (5′- CACGTTGTGCACCAGGAACT-3′)
Y-qp-F (5′-CACTAAATCTGATAACTTATTCCCCTATAAT-3′)
Y-qp-R (5′-AAAATTTTTTAATTTATTTAATTAACAAATATTG-3′)

## Results

### The F1 progeny of *mt: CoⅠ^ts^* females × *Het* males develop into adults, and the pupal eclosion rate correlates to the age of female flies

To study the transmission and functionality of paternal mitochondria in *D. melanogaster* and to construct an operational model to investigate the pathways regulating paternal transmission, we utilized 2 *D. melanogaster* lines, the *mt: CoⅠ^ts^* and the *Het* fly (Fig. 1a). The *mt: CoⅠ^ts^* is a temperature sensitive mutant line containing a point mutation in the mitochondrial gene *CoⅠ* (Hill *et al.* 2014). The *mt: CoⅠ^ts^* flies develop normally at a permissive temperature (18°C), but fail to eclosion from pupae at a restrictive temperature (29°C) (Fig. 1b). If eclosion is allowed to occur at the permissive temperature and then is followed by a shift to 29°C, the life span of the *mt: CoⅠ^ts^* flies is no more than 5 days (Hill *et al.* 2014). The *Het* fly contains 2 kinds of mitochondria, *D. melanogaster* mitochondrion (*mt:CoⅠ^ts^*, *mt: nd2^del1^*) and *D. yakuba* mitochondrion (Lieber *et al.* 2019).

We crossed *mt: CoⅠ^ts^* females with *Het* males and, surprisingly, found that the pupal eclosion rate of the F1 flies was greater than zero (ranging from 4.26 to 27.82%) at the restrictive temperature (Fig. 1c). Since there was a high pupal eclosion rate variance, we wondered if this correlated to the age of the female flies. By counting and comparing of the F1 eclosion rate of females of different ages, we found that the F1 eclosion rate decreased with the age of female flies. The finding that F1 flies were recovered strongly supports that paternal *D. yakuba* mitochondria are retained and functional. We also found that the majority of surviving F1 flies were females (91.65%). We do not have an explanation for the observation.

### Detection of paternal *D. yakuba* mtDNA in F1 flies from the cross *mt: CoⅠ^ts^* females × *Het* males

There have been several previous attempts to detect paternal mitochondrial leakage, including by analyzing interspecific crosses in *Drosophila* (Kondo *et al.* 1990, 1992; Dokianakis and Ladoukakis 2014; Polovina *et al.* 2020) and by examination of naturally occurring heteroplasmic populations (Nunes *et al.* 2013; Wolff *et al.* 2013). In our system, we detect the inheritance of paternal mitochondria in a simple way, by performing PCR to specifically detect paternal mtDNA in F1 flies. Primers are chosen to bind specifically to unique regions of mtDNA from either *D. melanogaster* or *D. yakuba* (Fig. 1a). The *D. yakuba* primers are specific and we did not detect any *D. yakuba* mtDNA in extracts from *mt: CoⅠ^ts^* flies (Fig. 2a).

We used this detection method to analyze YF1 flies (from the crosses of *mt: CoⅠ^ts^* × *Het*). PCR analysis revealed the presence of *D. yakuba* mtDNA fragments in YF1 flies, implying paternal *D. yakuba* mitochondria transmission (Fig. 2a). The PCR bands differ in intensity, implying the transmission efficiency among individual flies is variable, which may also contribute to the eclosion rate variability observed before. Sequencing results confirmed the identification of *D. yakuba* mtDNA sequences in YF1 flies (Fig. 2b).

Previous reports have shown that mtDNA can integrate into chromosomes to form NUMTs, which are mainly caused by chromosome breakage (Gray *et al.* 1999; Wei *et al.* 2020; Tigano *et al.* 2021). To exclude any effect of NUMTs, we prepared mitochondrial extracts and conducted PCR with the specific *D. yakuba* primers. As expected, *D. yakuba* fragments could be amplified from mitochondrial extracts, but not from nuclear extracts (Fig. 2c). Thus, the effect of NUMTs can be excluded. Moreover, qPCR analysis showed YF1 flies acquired about 5.85% *D. yakuba* mtDNA of *Het* flies (Fig. 2d). These results strongly suggest that YF1 flies inherited paternal *D. yakuba* mitochondria.

To further support that paternal *D. yakuba* mitochondria are inherited and functional, we conducted a Mitochondrial Respiratory Chain Complex Ⅳ Activity Assay on YF1 flies. We found that YF1 flies acquired about 45.67% complex Ⅳ activity of *Het* flies (Fig. 2e).

### Dysfunctional female mitochondria facilitate paternal *D. yakuba* mitochondrial transmission

Paternal mitochondrial leakage has been previously reported in *Drosophila* and *Mus musculus*. For example, 32–48% of paternal mitochondrial inheritance was reported in interspecific crosses of *Drosophila* (Sherengul *et al.* 2006). Other studies reported lower numbers, in one case 0.66% (Wolff *et al.* 2013) and in another 6% (Nunes *et al.* 2013). So far, there is no evidence that maternal mitochondria affect the inheritance of paternal mitochondria. In order to study the effect of maternal mitochondria, we conducted crosses and looked for evidence of paternal inheritance in the context of functional or dysfunctional maternal mitochondria.

First, we crossed *mt: CoⅠ^ts^* (female) with the *Het* fly (male) and cultured them at the permissive temperature of 18°C. The maternal mitochondria functioned normally and we could not detect paternal *D. yakuba* mitochondria by PCR analyses, indicating no or negligible paternal mitochondrial inheritance (Fig. 3a and c). Next, we crossed *w^1118^* (female) with *Het* fly (male) and cultured them at 29°C. The maternal mitochondria in this cross also functioned normally and we could not detect paternal *D. yakuba* mtDNA in F1 flies either (Fig. 3b and c). When we crossed *mt: CoⅠ^ts^* (female) × *Het* flies (male) at 29°C, where the maternal mitochondria in *mt: CoⅠ^ts^* were dysfunctional, the pupal eclosion rate ranged from 4.26 to 27.82% (Fig. 1c), suggesting successful paternal inheritance. These results suggest that the functionality of maternal mitochondria can affect the efficiency of paternal *D. yakuba* mitochondria elimination in fertilized eggs.

### mtDNA of paternal origin can be transmitted to subsequent generations

Our results show that paternal *D. yakuba* mitochondrial transmission can confer viability to offspring and rescue the mitochondrial respiratory chain complex Ⅳ activity in YF1 flies (Figs. 1c and 2e), indicating the rescue of mitochondrial function in YF1 flies. We then asked if paternal *D. yakuba* mitochondria could be prorogated in subsequent generations, although via the normal mode of maternal inheritance.

We collected YF1 females and backcrossed them with *mt: CoⅠ^ts^* flies (male), due to insufficient YF1 males. We found the pupal eclosion rate of YF2 (YF1 females × *mt: CoⅠ^ts^* male) flies at 29°C was about 12%, and not higher than that of YF1. This result could be explained by a dearth of paternal *D. yakuba* mitochondria in the YF1 fly reproductive system due to uneven distribution of paternal *D. yakuba* mitochondria in the first mitoses of YF1 embryos. Succeeding YF2 male and female flies were then collected, crossed, and cultured at 29°C for their life-time. Paternal *D. yakuba* mtDNA could be detected in YF3 and YF4 flies (Fig. 4a and b). The intensity of the PCR bands from YF1 flies was much lower compared to the *Het* flies (Fig. 2a), while the intensity of the PCR bands from YF3-4 flies was nearly identical to that of *Het* flies (Fig. 4a and b), suggesting the content of paternal mtDNA in YF3 and YF4 flies was proportionally higher than that in YF1 flies. Paternal mtDNA was also present in somatic cells and reproductive systems in YF3 flies (Fig. 4c).

A previous study showed that deleterious mitochondria are gradually replaced by healthy mitochondria (Hill *et al.* 2014). We therefore asked if the content of *D. yakuba* mtDNA changes after many generations. In order to answer this, we collected flies over 30 generations and then quantified the *D. yakuba* mtDNA content by qPCR. After 30 generations, the content of *D. yakuba* mtDNA increased identical to that of *Het* flies (Fig. 2d). Our results strongly demonstrate that paternally inherited mitochondria can be transmitted to subsequent generations just like normal mitochondria.

### The F1 progeny of *mt: CoⅠ^ts^* females with *D. melanogaster* strains can develop into adults

Since the *D. yakuba* mitochondria in *Het* flies are interspecific, it is possible that our result simply reflects the failure of elimination mitochondria in an interspecific cross. To investigate this possibility, we crossed *mt: CoⅠ^ts^* females with *w^1118^* males that contain wild-type *D. melanogaster* mitochondria (Supplementary Fig. 1a). The pupal eclosion rate of these WF1 flies was again greater than zero (ranging from 3.55 to 13.32%), though lower than the eclosion rate of YF1 flies from the cross of *mt: CoⅠ^ts^* (female) × *Het* fly (male) (Fig. 1c). To help rule out the effect of nuclear background, we crossed *mt: CoⅠ^ts^* females with another *D. melanogaster* strain Canton S (CS), and obtained a pupal eclosion rate of 19.91%±11.32% (Supplementary Fig. 1f).

We then looked for the presence of paternal mitochondrial DNA in the WF1 (from the cross of *mt: CoⅠ^ts^ ×w^1118^*) flies. The mitochondrial *CoⅠ* gene of *w^1118^* contains a XhoI restriction site, CTCGAG, whereas the restriction site is mutated to TTCGAG in the *mt: CoⅠ^ts^* mutants. DNA fragments amplified from *w^1118^* and *mt: CoⅠ^ts^* flies were treated with XhoI restriction enzyme. While XhoI treatment of the amplified DNA fragments from *w^1118^* yielded 2 small bands (230bp + 440 bp), the fragments from the *mt: CoⅠ^ts^* mutant fly did not (Supplementary Fig. 1b). XhoI treatment of the fragments of WF1 flies also failed to yield 2 small bands, which would have confirmed the presence of paternal mitochondrial DNA.

We considered that the amount of paternal mitochondria (with the XhoI restriction site) in WF1 flies may be below the detection limits. To gather support for this possibility, we mixed mt extracts from *mt: CoⅠ^ts^* and *w^1118^* flies (Supplementary Fig. 1c), PCR amplified the target fragments, and treated them with XhoI. At a ratio of 2:1 (*mt: CoⅠ^ts^*: *w^1118^*) the digested bands can be detected, but they are not detectable upon further dilution. This result supports the above possibility.

We used an alternative approach to investigate whether the WF1 generation contains paternal mtDNA, by measuring the Mitochondrial Respiratory Chain Complex Ⅳ activity in WF1 flies. Previous research has shown the cytochrome oxidase activity in *mt: CoⅠ^ts^* flies was unaffected at 18°C, but significantly reduced at the restrictive temperature of 29°C (Hill *et al.* 2014). We found that WF1 flies acquired about 40% complex Ⅳ activity compared to wild-type flies (Supplementary Fig. 1d).

Based on these results, we speculate that a similar phenomenon of paternal leakage exists in the F1 flies from crosses between *mt: CoⅠ^ts^* females and *D. melanogaster* strains containing wild type mitochondria, when maternal mitochondria are dysfunctional.

## Discussion

It is widely accepted that mitochondria are inherited maternally. Several pathways mediating paternal mitochondria elimination during spermatogenesis and zygote formation have been elucidated. However, paternal mitochondrial leakage has been reported in species such as fruit flies (Sherengul *et al.* 2006; Nunes *et al.* 2013), mice (Shitara *et al.* 1998), and sheep (Zhao *et al.* 2004). Heteroplasmic variants in patients with mitochondrial diseases in three independent families were reported in 2018, suggesting paternal mitochondrial inheritance in humans (Luo *et al.* 2018). Subsequently, paternal mitochondrial inheritance has become controversial. Some researchers believe that paternal mitochondrial leakage in interspecific hybridization is due to different genetic backgrounds, while others have proposed that a complex regulatory mechanism mediates paternal mitochondrial leakage. There are also those who believe that paternal mitochondrial leakage is a rare accident (Pagnamenta *et al.* 2021). Our results showed that exogenous paternal mitochondria can be transmitted to YF1 flies especially when maternal mitochondria are dysfunctional. It’s exciting to discover that the inherited mitochondria are functional and their levels increase in subsequent generations. Interestingly, we observed a similar phenomenon when only *melanogaster* mtDNA is present in both parents. Until we can definitively identify wild type *D. melanogaster* mtDNA in WF1 progenies, it remains highly speculative that paternal leakage occurs under the condition when maternal mitochondria are dysfunctional.

Nevertheless, we established a genetically tractable system for the study of paternal mitochondria leakage under any conditions. We envision that induced mutations under the genetic background of *mt: CoⅠ^ts^* would uncover novel pathways regulating paternal mitochondrial transmission.

## Supplementary Material

iyae014_Supplementary_Data

## Data Availability

*Drosophila* stocks are available upon request. The authors affirm that all data necessary for confirming the conclusions of the article are present within the article, figures. Supplemental material available at GENETICS online.
