# Supplementary material for: Maternal mitochondrial function affects paternal mitochondrial inheritance in Drosophila
Source: Genetics. 2024 Jan 30;226(4):iyae014. doi: 10.1093/genetics/iyae014 (PMC10990420; doi:10.1093/genetics/iyae014)
Supplement: iyae014_Supplementary_Data [file iyae014_supplementary_data.zip › Figure_S1_Legend_GENETICS-2024-306773.docx]

**Figure S1. The pupal eclosion rate in the cross of *mt:CoⅠ^ts^* (female) of different ages with *w^1118^* and CS males at 29°C and the paternal wild type mtDNA detection in F1 flies.**

1. The pupal eclosion rate of F1 flies from the cross *mt: CoⅠ^ts^* females (aged from 1-15 days) with *w^1118^* males. The results shown are mean ± SE. **B.** Paternal wild type mtDNA detection in F1 flies from the cross *mt: CoⅠ^ts^* females with *w^1118^* males. Fragments were amplified with primer F1 and R1 from the mtDNA of *w^1118^*, *mt: CoⅠ^ts^* and F1 flies. Amplified fragments were digested with XhoI restriction enzyme and subjected to DNA agarose gel electrophoresis. The amplified fragments (670bp) from the *mt: CoⅠ^ts^* mutant cannot be digested into two small bands, whereas the amplified fragments from *w^1118^* can be digested into two small bands (440+230bp). No digested small bands can be detected in F1 flies. **C.** DNA extracts from *mt: CoⅠ^ts^* and *w^1118^* flies were diluted (50:1, 25:1, 10:1, 5:1, 2:1, 1:1), amplified and then treated with XhoI restriction enzyme. At a 2:1 *mt: CoⅠ^ts^*, *w^1118^* dilution digested bands can be detected. **D.** Mitochondrial Respiratory Chain Complex Ⅳ Activity in *w^1118^*, *mt: CoⅠ^ts^* and F1 flies in 29°C. F1 flies acquire about 40% complex Ⅳ activity of *w^1118^* flies, significantly different from *mt: CoⅠ^ts^* flies (**, P<0.05). The results shown are mean ± SE. Three data sets were averaged. **E.** The pupal eclosion rate of CS and *mt: CoⅠ^ts^* males cross with *mt: CoⅠ^ts^* females. The results shown are mean ± SE.
