# Supplementary figures and images for: Maternal mitochondrial function affects paternal mitochondrial inheritance in Drosophila
Source: Genetics. 2024 Jan 30;226(4):iyae014. doi: 10.1093/genetics/iyae014 (PMC10990420; doi:10.1093/genetics/iyae014)

A

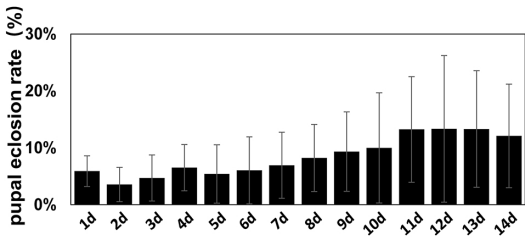

B

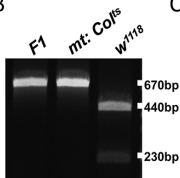

C

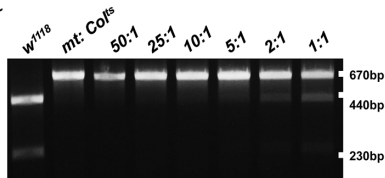

D

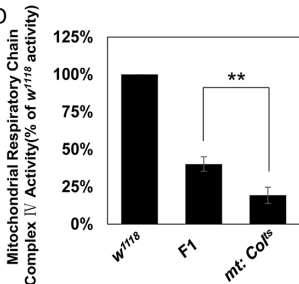

F

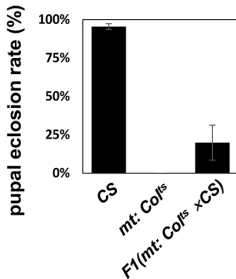

Supplement: iyae014_Supplementary_Data [file iyae014_supplementary_data.zip › Figure_S1_GENETICS-2024-306773.pdf]
